# Stimulation of insulin secretion by large-dose oral arginine administration in healthy adults

**DOI:** 10.3892/etm.2013.1119

**Published:** 2013-05-16

**Authors:** ZHU-QI TANG, TAO WU, SHI-WEI CUI, XIAO-HUI ZHU, TONG YIN, CUI-FANG WANG, JING-YI ZHU, AI-JUAN WU

**Affiliations:** 1 Department of Endocrinology, Affiliated Hospital of Nantong University, Nantong, Jiangsu 226001;; 2 Department of Nephrology, The Second Hospital of Shandong University, Jinan, Shandong 250033, P.R. China

**Keywords:** arginine, insulin, glucose tolerance

## Abstract

The effects of large-dose oral arginine administration on the secretion of insulin by islet β-cells in healthy adults were determined. Eight non-obese healthy volunteers with normal glucose tolerance participated randomly in tests with four stages (with an interval of at least 3 days): the 300 ml purified water stage (PWS), the 75 g glucose stage (GSS), the 30 g arginine stage (ARS) and the 75 g glucose with 30 g arginine stage (GAS). Venous blood samples were collected to detect the concentrations of glucose and insulin at baseline (0) and at 15, 30, 45, 60 and 120 min after drug administration. The glucose and insulin levels were steady in the PWS. The remaining three stages had similarly shaped insulin concentration-time curves, which differed from that of the PWS. The peak concentration of blood insulin and the net incremental area under the curve of blood insulin in the GSS, ARS and GAS were significantly higher compared with those in the PWS (P<0.05). In the ARS, the glucose levels remained stable; however, the net incremental area under the curve for blood insulin in the ARS was much lower compared with that in the GSS or GAS (P<0.05). Large-dose oral arginine administration may slightly stimulate insulin secretion by islet β-cells in healthy adults with normal glucose tolerance in a manner that is independent of glucose concentration.

## Introduction

The main pathogeneses of type 2 diabetes mellitus (T2DM) are insulin resistance and islet β-cell impairment, and the latter is central to the development and progression of T2DM (1,2). The extent and characteristics of islet β-cell dysfunction in the different courses of diabetes vary (3). Thus, the accurate evaluation of the insulin secretion level of islet β-cells in patients is important for the diagnosis, treatment and prognosis judgment of diabetes and for epidemiological analysis. Insulin secretion by islet β-cells is evaluated using glucose and non-glucose stimulation tests (4). If the islet β-cell is functionally damaged, then the pancreatic islet does not respond to glucose stimulation but does respond to non-glucose stimulation. If the damage progresses to organic damage, then the pancreatic islet does not respond to any stimulation (5,6).

Arginine is the most commonly used non-glucose stimulus. This amino acid has been widely studied and its intravenous administration has been shown to stimulate insulin secretion by islet β-cells (7,8). Thus, arginine administration has become a traditional method for the clinical evaluation of the insulin secretion capacity of islet β-cells (7,8). However, this method promotes insulin secretion non-physiologically since it avoids the effect of gut hormones on insulin secretion. Oral glucose administration stimulates the release of additional insulin compared with intravenous glucose administration in healthy adults (9). Similarly to glucose, arginine stimulates gut-affected insulin secretion through gut loading and promotes greater insulin release through oral rather than intravenous administration. Oral arginine administration may be used clinically in addition to oral glucose administration to evaluate the insulin secretion capacity of the islet β-cells of patients with T2DM. It may also be used to identify whether islet β-cell dysfunction or a reduced amount of β-cells in the pancreas of individuals with hyperglycemia is the cause of the damaged secretory capacity of islet β-cells, to ensure the appropriate diabetes treatment (10). Gannon *et al* reported that the serum insulin concentration in nine healthy subjects failed to increase following the oral administration of arginine with an average single dosage of 10.6 g (11). In a study conducted by Roslyn *et al,* six obese volunteers with >5 years of T2DM were administered oral arginine at 3 g/h for 10 h (total dosage, 30 g). The plasma concentrations of C-peptide and insulin over the 10 h administration period failed to increase (12). An insufficient dosage of arginine may be a reason for the two studies failing to obtain their prospective results (13). However, the stimulatory effect of large-dose oral arginine administration on insulin release remains unknown. In the present study, we investigated the effects of large-dose oral arginine on the secretion of insulin by islet β-cells in healthy subjects with normal glucose tolerance and used large-dose oral glucose administration as the control.

## Subjects and methods

### Subjects

Eight non-obese healthy volunteers (four males and four females) aged 20-40 years [mean ± standard deviation (SD), 30.5±3.7 years; Table I] with normal body mass indices (mean ± SD, 21.0±2.4 kg/m^2^) and normal glucose tolerance were enrolled in the study. These subjects took no regular medication and had no family medical history of diabetes. Subjects who suffered with diseases of the digestive system, heart, lung, liver and kidney, or thyroid dysfunction were excluded. Pregnant or lactating subjects and subjects who suffered from stress or infection were also excluded. This study was conducted in accordance with the Declaration of Helsinki and with approval from the Ethics Committee of the Affiliated Hospital of Nantong University. Written informed consent was obtained from all participants.

### Measurements

All volunteers participated randomly in tests with four stages (with an interval of at least 3 days): the 300 ml purified water stage (PWS), the 75 g glucose stage (GSS), the 30 g arginine stage (ARS) and the 75 g glucose with 30 g arginine stage (GAS), respectively. Participants received a low protein diet for 3 days. Afterwards, tests were conducted in a fasting state at 8:00 a.m. Glucose and/or arginine were consumed with 300 ml purified water in 5 min. Venous blood samples were collected from each subject at baseline (0) and at 15, 30, 45, 60 and 120 min after drug administration to detect the serum concentrations of glucose and insulin. The discomfort reactions of the subjects during the test period and the following 3 days were recorded.

### Data analysis

The area under the concentration-time curve (AUC) was calculated by the trapezoidal area formula (13). The net incremental area was AUC minus the area of the fasting plasma glucose or fasting insulin. The relative percentage of the net incremental area was calculated as [(AUC_N_ - AUC_G_)/AUC_G_] ×100, where AUC_N_ represents the AUC of a certain stage and AUC_G_ is the AUC of the GSS.

### Statistical analysis

All statistical analyses were performed using SPSS 17.0 for Windows. Data are presented as mean ± SD for the parameters in Gaussian distribution. Otherwise, the median (quartile) [m(Q_L_,Q_U_)] were used. The serum concentrations of glucose and insulin between different stages were analyzed by repeated measurement variance analysis followed by Tukey’s multiple comparison test. The AUCs of the four stages were compared by the rank sum test. P<0.05 was considered to indicate a statistically significant difference.

## Results

### Blood glucose concentration

**Figure 1.**
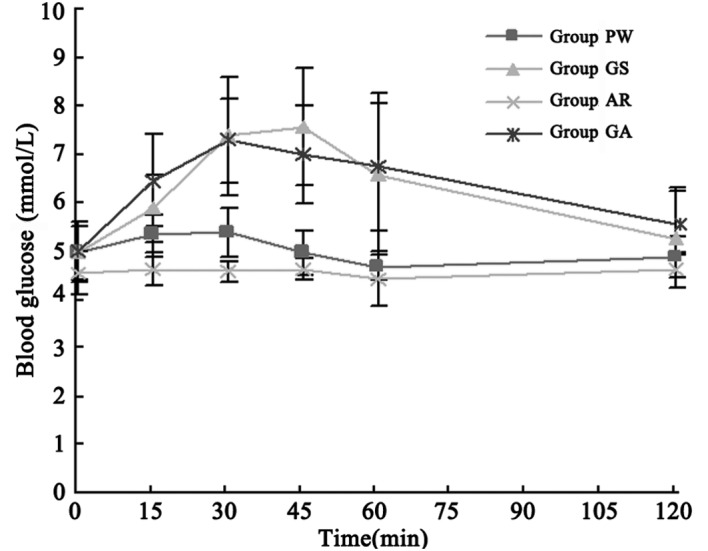
Blood glucose concentration curves of the four stages. Venous blood samples were collected at baseline (0) and at 15, 30, 45, 60 and 120 min after drug administration to detect the serum glucose concentrations by the glucose oxidase method. PW, purified water; GS, glucose; AR, arginine; GA, glucose with arginine.

The venous blood samples collected at baseline (0) and at 15, 30, 45, 60 and 120 min after drug administration were used to detect the serum concentration of glucose through the glucose oxidase method. There was no significant difference of blood glucose concentration at baseline (0 min) among four stages (Table II; P>0.05). The blood glucose level during the detection period was not reduced significantly in the ARS compared with that in the PWS (P>0.05). The glucose concentration-time curve of the GSS was similar to that of the GAS (Fig. 1). All samples were run in duplicate.

### Blood insulin concentration

**Figure 2.**
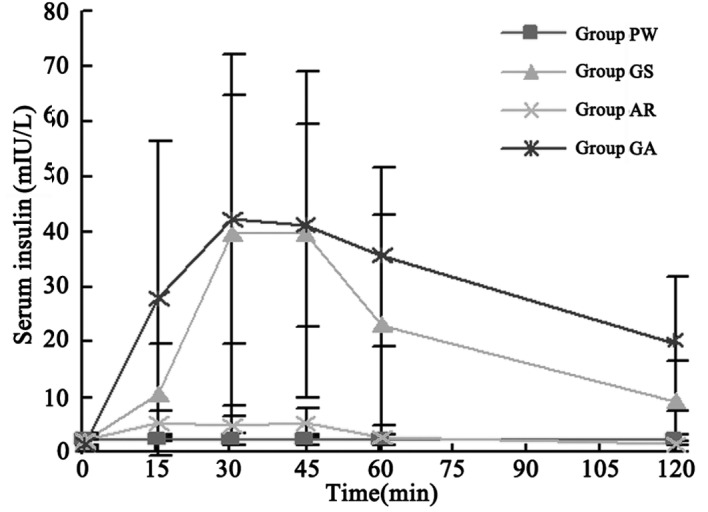
Blood insulin concentration curves of the four stages. Samples collected at baseline (0) and at 15, 30, 45, 60 and 120 min after drug administration were used to detect the insulin concentrations by a chemoluminescence test. PW, purified water; GS, glucose; AR, arginine; GA, glucose with arginine.

The venous blood samples collected were also used to detect the serum concentrations of insulin by a chemoluminescence test and were run in duplicate. At baseline (0 min), no significant difference was identified among four stages (Table III; P>0.05). Insulin concentration in the PWS at each time point after administration demonstrated no significant difference compared with that of the baseline (P>0.05). However, the peak concentration in the ARS following administration was markedly higher compared with that of the baseline (P<0.05); however, it was still significantly lower compared with those of the GSS and GAS (P<0.05). The insulin concentration curves of the GSS, ARS and GAS were all similarly shaped (Fig. 2).

### Concentration-time curve

The net incremental AUCs of glucose (AUC_g_) and insulin (AUC_i_) in the GSS and GAS increased markedly compared with those of the PWS and ARS (P<0.05). AUC_i_ in the ARS increased notably compared with that in the PWS (P<0.05); however, the difference in AUC_g_ between the ARS and PWS was not significant (P>0.05). In Table IV, the AUC_g_ and AUC_i_ in the GSS and GAS demonstrated no significant differences (P>0.05). The AUC_g_ and AUC_i_ in the GSS and ARS compared with those in the GAS were not significantly different (z=−0.42, P=0.67; z=−1.26, P=0.21).

### Tolerability

Arginine tastes bitter, salty and acerbic. Fourteen of the 16 administrations resulted in nausea but not vomiting. Following all 16 administrations, the subjects had diarrhea three to five times without abdominal pain on the day of administration and the worst case lasted for seven episodes of diarrhea. All subjects fully recovered on the second day.

## Discussion

A previous study reported that oral protein increases insulin concentration (14). Another study demonstrated that amino acids absorbed by the gut stimulate insulin release and arginine may be the main functional component (15). Floyd *et al* first identified that intravenous arginine administration increases the insulin concentration in the blood circulation (16). Thus, the intravenous arginine load test is clinically used as the non-glucose promoting secretion test to evaluate the function of islet β-cells in patients with T2DM (17-20), leading to the exploration of the oral arginine load test.

In 2002, Gannon *et al* performed the oral arginine load test and identified that oral arginine increases glucagon levels and delays glucose processing without affecting the gastric emptying time. However, this amino acid does not increase the serum insulin concentration (11). Given that the test was conducted on healthy adults and obese volunteers with T2DM by low-dose oral arginine administration, the blood arginine concentration was much lower than the oral sensitivity threshold. In individuals with normal glucose levels, the serum arginine concentration is 0.7 mmol/l when the insulin secretion volume reaches half of the effective dose (ED_50_) in phase one, and the insulin secretion volume is 2.7 mmol/l in phase two (13). Therefore, the large-dose oral arginine administration may stimulate insulin secretion as effectively as glucose; this possibility was the motivation for our study.

Previous oral arginine load tests have shown that arginine is perfectly tolerated in all subjects (11,12). However, in the present study, the administration of a large dose of oral arginine resulted in nausea in 14 out of 16 cases and all subjects experienced varying degrees of diarrhea, which indicates that the arginine dose used had reached the maximum tolerance for a single oral dosage. Our results demonstrated that large-dose oral arginine administration has the same effect on glucose concentration as treatment with purified water. Large-dose oral arginine administration stimulates insulin release independent of glucose concentration; however, the extent is much less compared with that of intravenous administration (8,21). The stimulating effect of oral and intravenous glucose on insulin release is not observed in arginine administration (9). This observation may be related to the low bioavailability of ∼21% of oral arginine (13). Oral arginine administration is affected by the gastrointestinal digestion absorption rate, arginine metabolism of the intestinal mucosa cells, the first-pass removal rate of the liver and others (22). Intravenous administration avoids the metabolism of the gastrointestinal system and liver, as well as effectively increasing the arginine content in the blood circulation to significantly stimulate islet β-cells. In addition, oral arginine administration has no stimulating effect on gut-affected insulin secretion the same with oral glucose administration (23). Arginine combined with glucose quickly stimulates insulin secretion and persists for a long time; however, it has no synergistic stimulatory effect (19,24). Promoters of insulin secretion are divided into two classes, namely, the initiator or primary irritant and the enhancer or secondary irritant. The initiator increases insulin release without any other irritant. Glucose is the most effective initiator. The enhancer does not have an effect by itself; however, it promotes insulin secretion with the existence of an initiator, including glucose (25). Arginine may have a stronger stimulatory effect on insulin secretion as a secondary irritant than as a primary irritant. Therefore, oral arginine load tests may be applied in patients with impaired glucose tolerance. A different set of subjects are required for these tests to be conducted. Further studies are required to detect the concentration of serum arginine, glicentin and incretin.

In conclusion, the current study indicates that a large dose of oral arginine does not affect the glucose concentration and is not able to effectively stimulate the insulin secretion of healthy adults with normal glucose tolerance. Further studies are required to determine whether a large dose of oral arginine stimulates insulin secretion in patients with serious sugar toxicity and suffering from impaired glucose tolerance or damaged islet cells that are unresponsive to glucose stimulation. Further improvements, including changing the form of medication, buccal and sublingual administration to avoid the metabolism of the gastro-intestine, or using a microcapsule package technique to reduce the removal rate of the liver, may promote the clinical application of other administration means of arginine stimulation tests as alternatives to intravenous administration.

## Figures and Tables

**Table I.** Clinical characteristics of the subjects.

Characteristics before test (n=8)	
Gender, male N (%)	4 (50)
Age, years	30.5±3.7
Weight, kg	56.8±6.8
BMI, kg/m^2^	21.0±2.4
Fasting serum glucose concentration, mmol/l	4.8±0.6
Fasting insulin concentration, mIU/l	1.79±1.2

BMI, body mass index. Data are mean ± standard deviation (SD).

**Table II.** Blood glucose concentration in the four stages (mmol/l) at different time points.

Stage	0 min	15 min	30 min	45 min	60 min	120 min
PWS	5.1 (4.5, 5.5)	5.4 (5.0, 5.7)	5.4 (5.0, 5.8)	5.0 (4.6, 5.3)	4.7 (4.4, 4.9)	4.9 (4.5, 5.2)
GSS	4.8 (4.5, 5.4)	6.2 (5.3, 6.5)^a^	7.3 (6.4, 8.4)^a^	7.3 (6.5, 8.6)^a^	6.2 (5.2, 8.0)^a^	5.5 (4.4, 6.1)
ARS	4.6 (4.2, 4.9)	4.7 (4.3, 4.9)	4.6 (4.4, 4.7)	4.7 (4.4, 4.8)	4.5 (3.9, 4.9)	4.6 (4.3, 4.9)
GAS	5.0 (4.5, 5.5)	6.4 (5.7, 7.2)^a^	7.1 (6.5, 8.0)^a^	6.6 (6.1, 7.8)^a^	6.6 (5.6, 7.8)^a^	5.7 (5.0, 6.1)^a^

Data are presented as median (quartile) [m(Q_L_, Q_U_)].

a P<0.05 vs. PWS and ARS values. PWS, purified water stage; GSS, glucose stage; ARS, arginine stage; GAS, glucose with arginine stage.

**Table III.** Serum insulin concentration in the four stages (mIU/l) at different time points.

Stage	0 min	15 min	30 min	45 min	60 min	120 min
PWS	2.29 (1.44, 3.13)	2.20 (1.68, 2.78)	2.51 (1.59, 3.18)	2.23 (1.37, 2.97)	1.90 (1.46, 2.86)	2.06(1.45, 2.80)
GSS	1.28 (0.47, 3.22)	6.77 (3.08, 18.01)	30.18 (11.88, 66.92)^b^	33.15 (14.95, 64.33)^b^	16.76 (6.30, 39.82)^b^	8.97(2.83, 15.40)^b^
ARS	1.91 (1.20, 2.97)	4.89 (3.20, 7.05)	3.60 (1.94, 7.60)	4.15 (2.89, 7.56)^a^	2.11 (0.49, 4.56)	0.74(0.22, 2.63)
GAS	1.33 (0.73, 2.12)^c^	15.66 (3.71, 51.74)^b^	39.36 (23.44, 60.95)^b^	32.19 (25.63, 56.57)^b^	34.88 (21.91, 49.11)^b^	16.34 (9.32, 29.90)^b^

Data are presented as median (quartile) [m(Q_L_, Q_U_)].

a P<0.05 vs. baseline value;

b P<0.05 vs. ARS value;

c P<0.05 vs. 120 min value. PWS, purified water stage; GSS, glucose stage; ARS, arginine stage; GAS, glucose with arginine stage.

**Table IV.** Net incremental area under the concentration-time curve of glucose and insulin and its relative percentage in the four stages.

Stage	AUC_g_ [(mmol/l) x min]	AUC_i_ [(mIU/l) x min]	AUC_g_/AUC_g_ (%)	AUC_i_/AUC_i_ (%)
PWS	−3.5 (−61.5, 54.6)	−10.7 (−66.1, 44.8)	−145.0 (−136.1, −94.7)	−109.5 (−114.3, −87.5)
GSS	161.3 (42.8, 279.8)^a^^b^	2274.1 (941.0, 3607.3)^a^^b^	100	100
ARS	−1.4 (−53.7, 50.9)	129.7 (−8.1, 267.5)^b^	−40.5 (−123.5, −90.6)	−342.6 (−110.7, −80.4)
GAS	167.3 (91.8, 242.7)^a^^b^	3425.2 (2502.8, 4347.5)^a^^b^	281.0 (−2.2, 9.7)	227.8 (141.6, 312.1)

AUC_g_, the net incremental area under the glucose concentration-time curve; AUC_i_, the net incremental area under the insulin concentration-time curve. Data are presented as median (quartile) [m(Q_L_, Q_U_)].

a P<0.05 vs. ARS value;

b P<0.05 vs. PWS value. PWS, purified water; GSS, glucose; ARS, arginine; GAS, glucose with arginine.
